# Effects of Perioperative Recombinant Human Brain Natriuretic Peptide in Patients Undergoing Cardiac Surgery: A Systematic Review and Meta-Analysis

**DOI:** 10.31083/RCM36423

**Published:** 2025-09-18

**Authors:** Juanjuan Shao, Liangshan Wang, Chengcheng Shao, Yan Wang, Jin Li, Jianfeng Luo, Zhongtao Du, Xiaotong Hou

**Affiliations:** ^1^Center for Cardiac Intensive Care, Beijing Anzhen Hospital, Capital Medical University, 100029 Beijing, China; ^2^Department of Biostatistics, School of Public Health, Fudan University, 200032 Shanghai, China

**Keywords:** natriuretic peptides, perioperative medicine, cardiac surgical procedures, meta-analysis

## Abstract

**Background:**

Clinical trials have demonstrated the efficacy of recombinant human brain natriuretic peptide (rhBNP) in managing acute decompensated heart failure. Moreover, since rhBNP performs roles in hemodynamic regulation, neurohormonal balance, and renal function, rhBNP administration could benefit cardiac surgery patients. We conducted a systematic review and meta-analysis to evaluate the impact of perioperative rhBNP in cardiac surgery patients.

**Methods:**

We conducted a comprehensive search of the MEDLINE, Embase, the Cochrane Library, CNKI, and WANFANG databases from January 1, 2007, until December 31, 2023, identifying randomized controlled trials (RCTs) that examined the use of rhBNP during cardiac surgery. We estimated the treatment effects of perioperative rhBNP administration using a random-effects meta-analysis. The primary cardiovascular endpoint was the change in left ventricular ejection fraction (LVEF); meanwhile, renal function was assessed using the 24-hour urine output, changes in estimated glomerular filtration rate (eGFR), and serum creatinine (SCr) levels. Additional parameters included pulmonary artery pressure (PAP), adverse event (AE) incidence, respiratory support duration, ICU length of stay (ICU LOS), hospital length of stay (hospital LOS), and tumor necrosis factor-α (TNF-α) levels.

**Results:**

Our meta-analysis included 38 RCTs encompassing 2280 patients. The use of rhBNP in the perioperative period significantly enhanced LVEF compared to the control group (mean difference = 3.13 (95% CI [1.88, 4.37]; *p* < 0.00001). Additionally, rhBNP administration was associated with a significant increase in the 24-hour urine volume (mean difference = 401.42, 95% CI [253.06, 549.77]; *p* < 0.00001) and an improvement in eGFR (mean difference = 13.94, 95% CI [5.57, 22.31]; *p* = 0.001). Meanwhile, perioperative administration of rhBNP significantly reduced SCr levels (mean difference = –14.55, 95% CI [–22.04, –7.06]; *p* < 0.0001). In addition, rhBNP significantly decreased PAP, the incidence of AEs, the duration of respiratory support, ICU LOS, hospital LOS, and TNF-α levels.

**Conclusions:**

These findings underscore the potential benefits of rhBNP as a perioperative treatment in patients undergoing cardiac surgery.

## 1. Introduction

Cardiovascular diseases are widespread and significantly contribute to the global burden of morbidity and mortality. Cardiac surgery (CS), a mainstay therapeutic approach for a variety of heart conditions, often necessitates the use of cardiopulmonary bypass (CPB). However, despite significant medical and surgical advancements, postoperative complications and mortality rates after CS remain a concern, and are influenced by the type of surgery, existing patient comorbidities, and the overall health status of patients [1].

CS encompasses a range of procedures, including coronary artery bypass grafting (CABG), valve repair or replacement, and the correction of congenital heart defects. Patients typically exhibit poor preoperative cardiac reserve functions before cardiac surgery [1, 2]. Moreover, surgical trauma, intraoperative myocardial ischemia, and cardiopulmonary bypass (CPB) pose a risk of myocardial ischemia and ischemia-reperfusion injury, potentially resulting in low cardiac output syndrome [3, 4]. Hence, enhancing the safeguarding of cardiac functions and preventing myocardial damage in patients undergoing cardiac surgery remains crucial.

Brain natriuretic peptide (BNP), synthesized predominantly in the ventricles of the heart in response to increased pressure and volume, plays a crucial role in blood pressure regulation and fluid balance. Indeed, BNP exhibits vasodilatory effects, promotes natriuresis (sodium excretion in urine), and hinders the release of renin and aldosterone [5, 6].

The recombinant human B-type natriuretic peptide (rhBNP) closely mirrors the structure and biological activity of the endogenous BNP and contributes to reducing blood pressure, alleviating fluid overload, and reducing the workload of the heart [7]. Approved by the US FDA in 2001 for treating acute decompensated heart failure (ADHF), rhBNP has been in clinical use in China since 2005. Subsequently, extensive trials have confirmed its safety and efficacy in managing congestive heart failure [8, 9], meaning its application has been extended to the perioperative period of CS [9, 10].

Some trials have shown that rhBNP positively affects heart functions following cardiac surgery [11, 12, 13, 14, 15, 16, 17, 18, 19, 20, 21, 22, 23, 24, 25, 26, 27, 28, 29, 30, 31, 32, 33, 34, 35, 36, 37, 38, 39, 40, 41, 42, 43]. However, in other trials, the effects of rhBNP were not observed [44, 45, 46, 47, 48]. A systematic review by Hua *et al*. [49] analyzed randomized controlled trials (RCTs) from 2007 to 2016, focusing on the impact of rhBNP on patient outcomes following CS. The meta-analysis by Hua *et al*. [49] indicated that the perioperative use of rhBNP was safe and effective, and improved patient prognoses.

Therefore, this study aimed to update prior systematic reviews for the following reasons: (1) New trials have been conducted over more than 5 years since the meta-analysis by Hua *et al*. [49], and (2) that meta-analysis was limited to patients undergoing extracorporeal circulation procedures rather than the whole scope of cardiac surgery. (3) The reported outcomes of the previous meta-analysis by Hua were limited, including adverse events, mortality rates after the surgery, ICU LOS, hospital LOS, 24-hour urine volumes after the surgery, and changes in serum creatinine (SCr) and TNF-α levels. Consequently, our review broadens the scope to encompass a comprehensive range of cardiac surgeries to incorporate a more extensive array of outcomes. Additionally, the current dataset allows for in-depth subgroup analyses of specific procedures, such as coronary artery bypass grafting (CABG) and valve surgeries, offering a nuanced perspective on the efficacy of rhBNP in different surgical contexts.

## 2. Materials and Methods 

### 2.1 Literature Retrieval

We conducted a systematic search for RCTs that assessed the use of perioperative rhBNP in cardiac surgery patients. This search encompassed English-language databases: Medline, Embase, and the Cochrane Library; Chinese-language databases, including CNKI and WANFANG. Our retrieval spanned from January 1, 2007, to December 31, 2023. The literature search strategy included a comprehensive list of all terminology variants referring to rhBNP, a 32-amino acid peptide identical to endogenous BNP in structure and function. Keywords in English focused on terms such as “brain natriuretic peptide”, “nesiritide”, and “cardiopulmonary bypass”, while Chinese searches included “rhBNP”, “nesiritide”, and “Xinhuosu”, among others. We performed a retrospective review of the identified literature.

### 2.2 Inclusion and Exclusion Criteria

We included trials with the following characteristics:

(1) Patients undergoing (non-interventional) cardiac surgery, (2) perioperative use of rhBNP vs. no rhBNP use, (3) baseline treatments that included a placebo (normal saline, Ringer’s solution, etc.) or usual treatment, (4) evaluation of cardiac function (left ventricular ejection fraction (LVEF), etc.), kidney function (SCr levels) or ICU LOS stay, hospital LOS, mortality, (5) was a RCT.

Studies were excluded for unclear intervention strategies, duplication, lack of defined outcomes, or inability to contribute data to the analysis.

### 2.3 Literature Selection and Quality Evaluation

Two reviewers independently performed the study screenings and quality evaluations, extracting information according to a pre-designed data collection form. Discrepancies were resolved through discussion or, if necessary, by consultation with a third reviewer. The included trials were evaluated using the Cochrane risk of bias judgement system.

### 2.4 Outcomes

Outcomes of efficacy analyzed in this systematic review included LVEF, 24-hour urine volumes, the maximal changes in eGFR, SCr levels, and PAP.

Outcomes of safety included the incidence of AEs and the mortality rate. Mortality rate was defined as death during rhBNP infusion or within the entire hospital stay, as reported in those manuscripts.

### 2.5 Statistical Analysis

We utilized Review Manager version 5.2 software (RevMan for Windows 2003; the Nordic Cochrane Center, Copenhagen, Denmark) for statistical analysis. Heterogeneity was assessed using the I^2^ test, with a threshold of *p* > 0.1 and I^2^ ≤ 50% considered to indicate low heterogeneity. The random-effects model was employed regardless of heterogeneity due to its conservative nature. The subgroup analysis by CABG and valve procedure was conducted to explore the consistency of results. A sensitivity analysis of excluding particular studies was used to examine the robustness of the synthesized results. Risk ratios (RRs) were employed to analyze count data. Continuous data were assessed using weighted mean differences (WMDs). All analyses were accompanied by 95% confidence intervals (CIs). Statistical significance for pooled effect estimates (RR and WMD) was defined as a two-sided *p*-value < 0.05.

## 3. Results 

### 3.1 Study Characteristics 

**Fig. 1.**
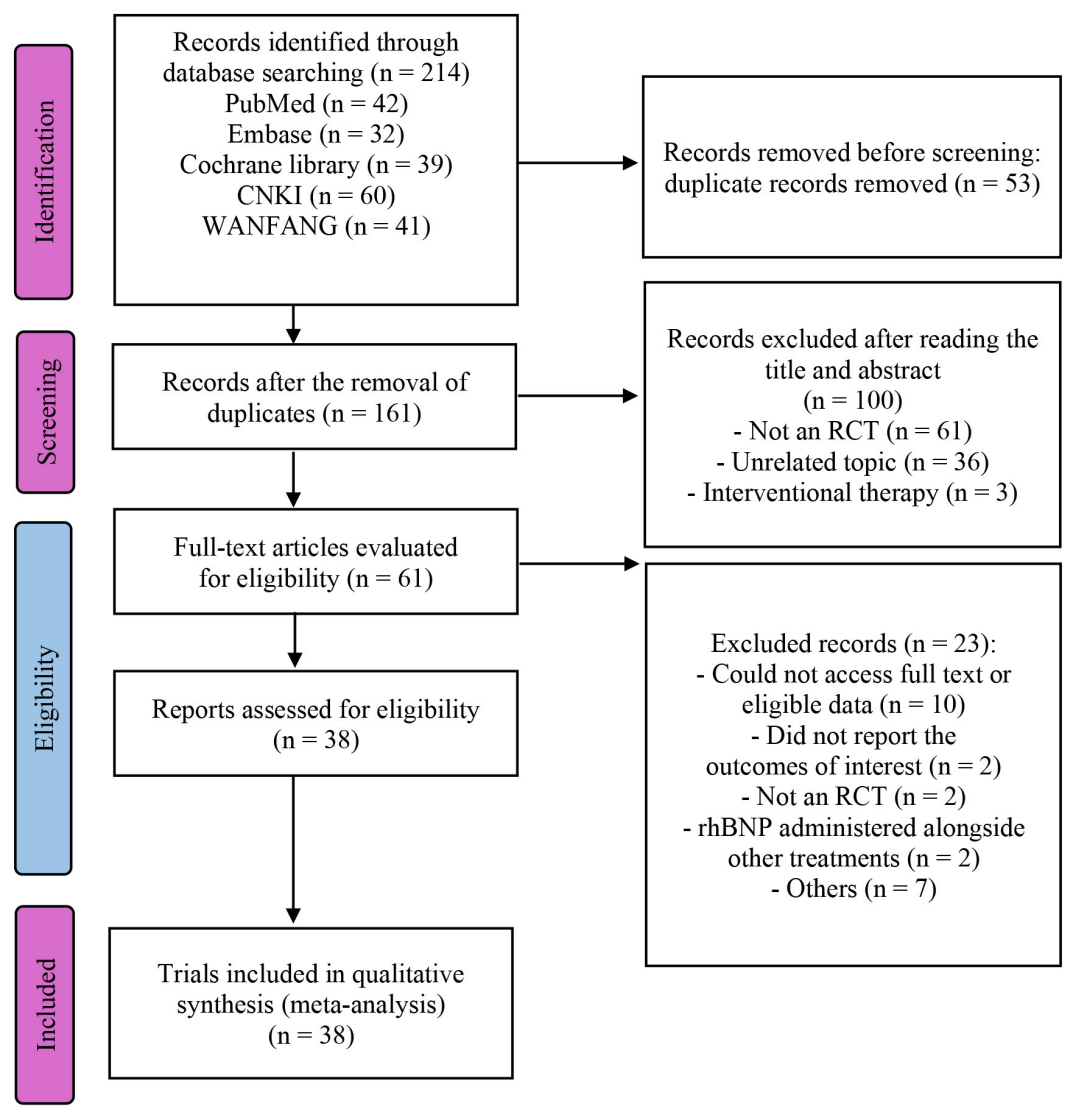
**PRISMA flow diagram of the study process**. PRISMA, Preferred Reporting Items for Systematic reviews and Meta-Analyses; RCT, randomized controlled trial; rhBNP, recombinant human brain natriuretic peptide.

A total of 214 trials were identified following the preliminary screening. After excluding duplicates and non-eligible studies, and following a thorough review of full texts and references, 38 RCTs encompassing 2280 participants were selected for detailed analysis [11, 12, 13, 14, 15, 16, 17, 18, 19, 20, 21, 22, 23, 24, 25, 26, 27, 28, 29, 30, 31, 32, 33, 34, 35, 36, 37, 38, 39, 40, 41, 42, 43, 44, 45, 46, 47, 48]. Fig. 1 provides a visual representation of the publication selection process and its outcomes; meanwhile, **Supplementary Table 1** offers a concise overview of the included trials and an assessment of the methodologies employed.

### 3.2 Risk of Bias Assessment

**Fig. 2.**
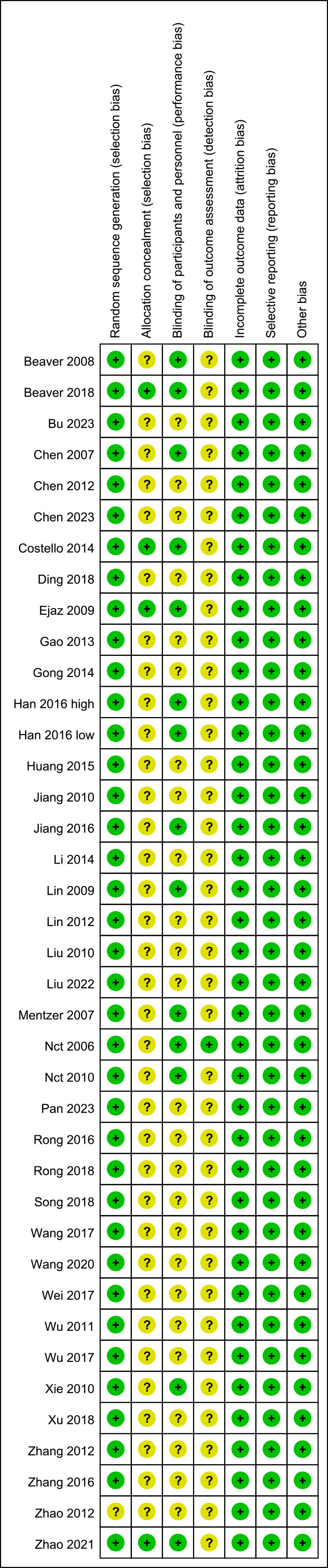
**Risk of bias map**.

Two independent reviewers assessed the risk of bias in the studies using the Cochrane risk of bias map. As is shown in Fig. 2, the studies had little bias in randomization, incomplete data, and selection reporting. Meanwhile, a moderate level of bias existed in allocation concealment and blinding.

## 4. Meta-Analysis Results

### 4.1 Left Ventricular Ejection Fraction

**Fig. 3.**
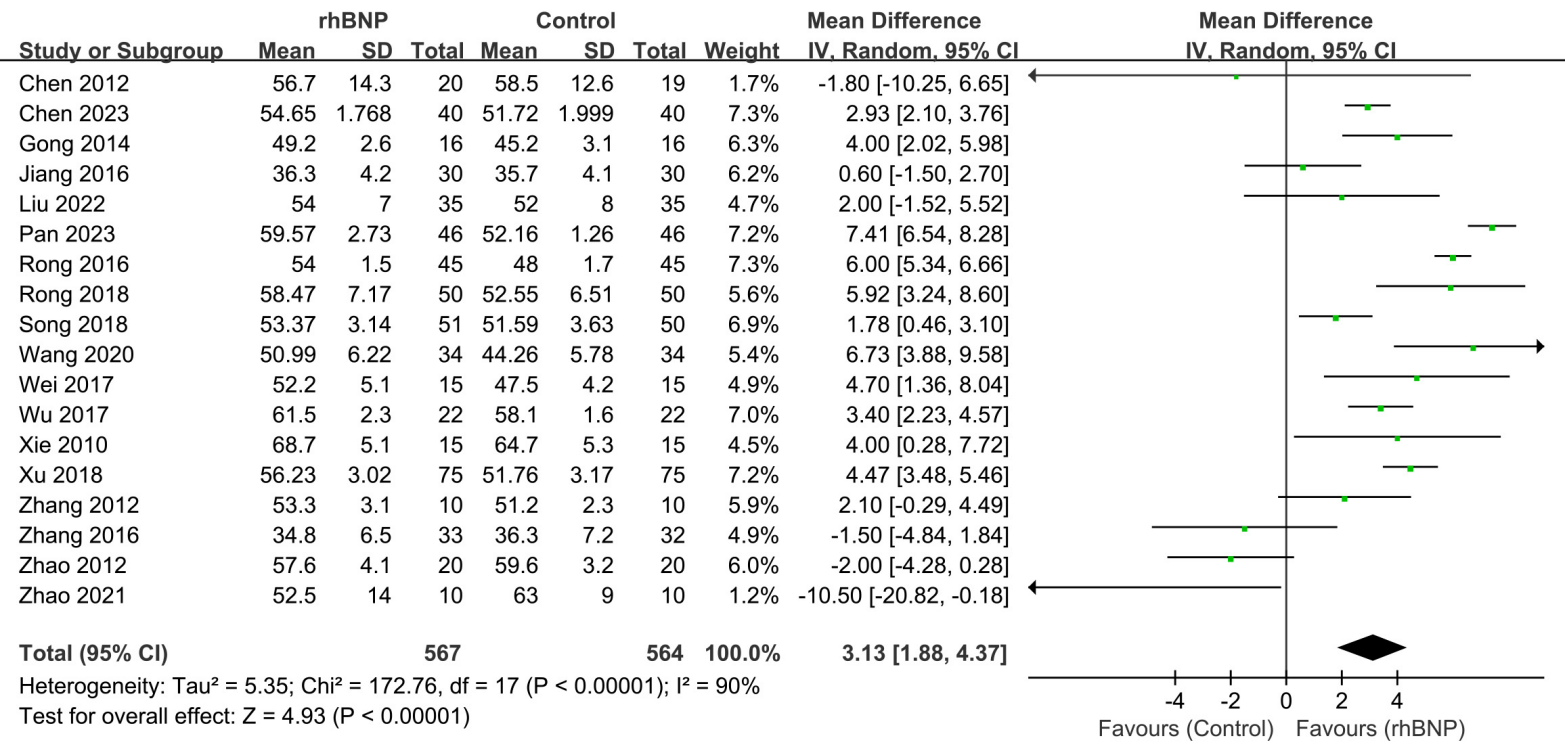
**Comparison of left ventricular ejection fraction (LVEF)**. rhBNP, recombinant human brain natriuretic peptide; SD, standard deviation; df, degrees of freedom.

Eighteen trials presented the maximum changes in LVEF post-surgery (Fig. 3). All data displayed high heterogeneity (I^2^ = 90%; *p* < 0.00001). The meta-analysis indicated a significant increase in LVEF among patients receiving rhBNP compared to the control group (mean difference = 3.13, 95% CI [1.88, 4.37]; *p* < 0.00001).

LVEF improvement was also found in the subgroup analysis within patients using CABG (**Supplementary Fig. 1**) and valve procedure (**Supplementary Fig. 2**), with a mean difference in LVEF and 95% CI of 3.51 (1.19, 5.83) and 2.05 (–1.88, 5.98), respectively.

The high heterogeneity seemed mainly induced by Zhao’s study [39]. The non-significant LVEF improvement in the valve surgery subgroup (mean difference = 2.05, 95% CI [–1.88, 5.98]; *p* = 0.31; I^2^ = 72%) was primarily driven by the baseline LVEF imbalance in Zhao (2021). Therefore, upon excluding Zhao (2021) (**Supplementary Fig. 3**), the valve surgery subgroup analysis showed: I^2^ decreased from 72% to 53% and statistically significant LVEF improvement with mean difference = 4.41 (95% CI [1.56, 7.25]; *p* = 0.002).

### 4.2 24-Hour Urine Volumes

**Fig. 4.**
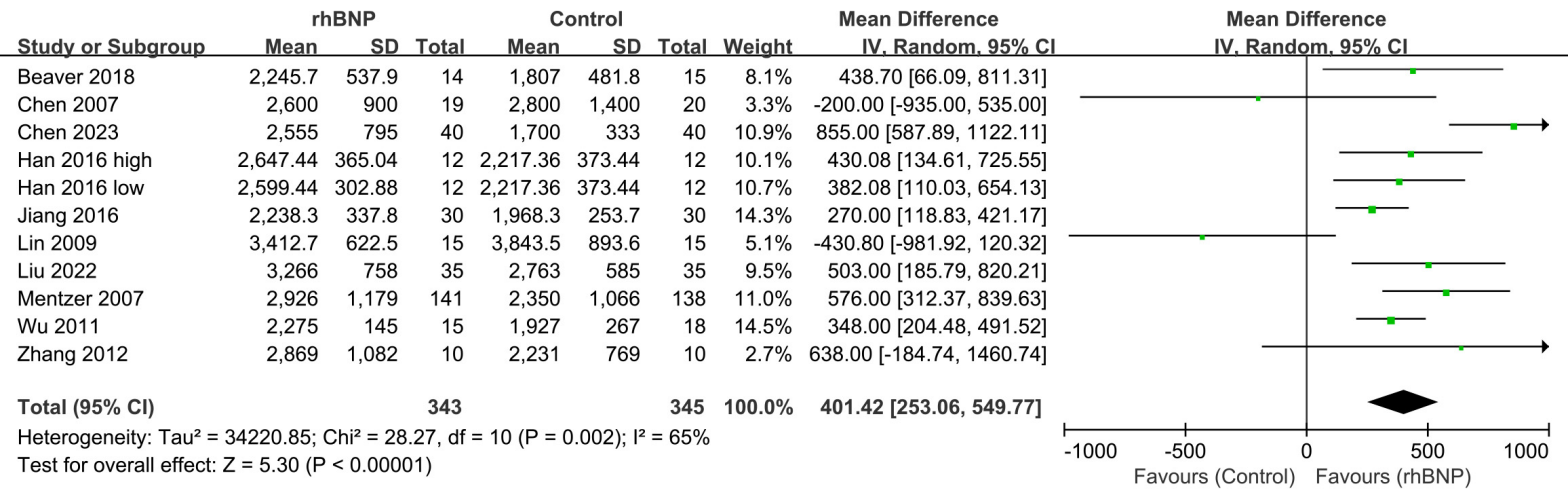
**Comparison of 24-hour urine volumes**. rhBNP, recombinant human brain natriuretic peptide; SD, standard deviation; df, degrees of freedom.

Eleven trials reported findings regarding the 24-hour urine volumes post-surgery (Fig. 4). A random-effects model was utilized because of the moderate heterogeneity observed across all trials (I^2^ = 65%; *p* = 0.002). The meta-analysis revealed that the 24-hour urine output post-surgery was significantly greater in the rhBNP group than in the control group (mean difference = 401.42, 95% CI [253.06, 549.77]; *p* < 0.00001).

Increased 24-hour urine volume post-surgery was also found in the subgroup analysis within patients using CABG (**Supplementary Fig. 4**) and valve procedure (**Supplementary Fig. 5**), with a mean difference and 95% CI of 581.77 (330.71, 832.83) and 295.08 (3.09, 587.08), respectively.

### 4.3 Maximal Changes in eGFR

**Fig. 5.**
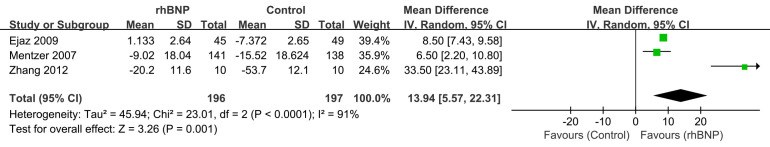
**Comparison of estimated glomerular filtration rate (eGFR)**. rhBNP, recombinant human brain natriuretic peptide; SD, standard deviation; df, degrees of freedom.

Three trials reported the maximum changes in eGFR post-surgery (Fig. 5). All data exhibited high heterogeneity (I^2^ = 91%; *p* < 0.0001). The meta-analysis revealed a significant improvement in eGFR for the rhBNP group compared to the control group (mean difference = 13.94, 95% CI [5.57, 22.31]; *p* = 0.001).

### 4.4 Serum Creatinine Levels

**Fig. 6.**
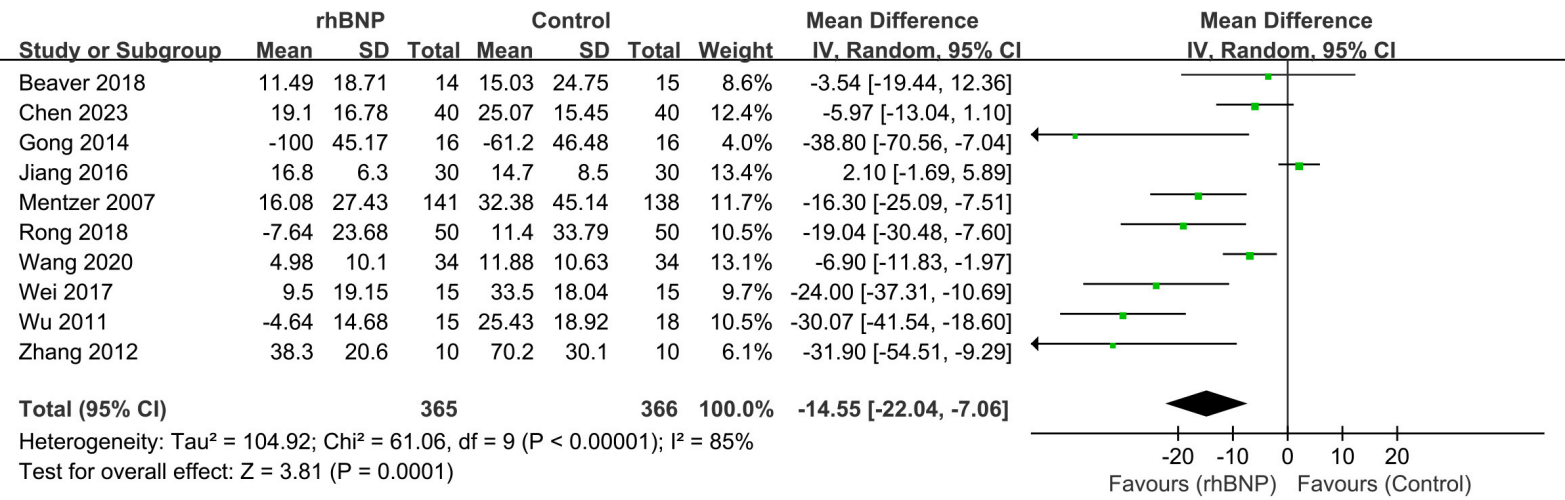
**Comparison of serum creatinine (SCr) levels**. rhBNP, recombinant human brain natriuretic peptide; SD, standard deviation; df, degrees of freedom.

Ten trials reported maximum changes in the SCr levels post-surgery (Fig. 6). All data exhibited high heterogeneity (I^2^ = 85%; *p* < 0.00001). The meta-analysis indicated a significant decrease in SCr levels in the rhBNP group compared to the control group (mean difference = –14.55, 95% CI [–22.04, –7.06]; *p* = 0.0001).

A notable significant reduction in maximum postoperative SCr changes was observed in the treatment group, particularly among CABG patients (**Supplementary Fig. 6**), as compared to the control group (mean difference = –17.67, 95% CI [–20.83, –14.51]; *p* < 0.00001).

### 4.5 PAP

**Fig. 7.**
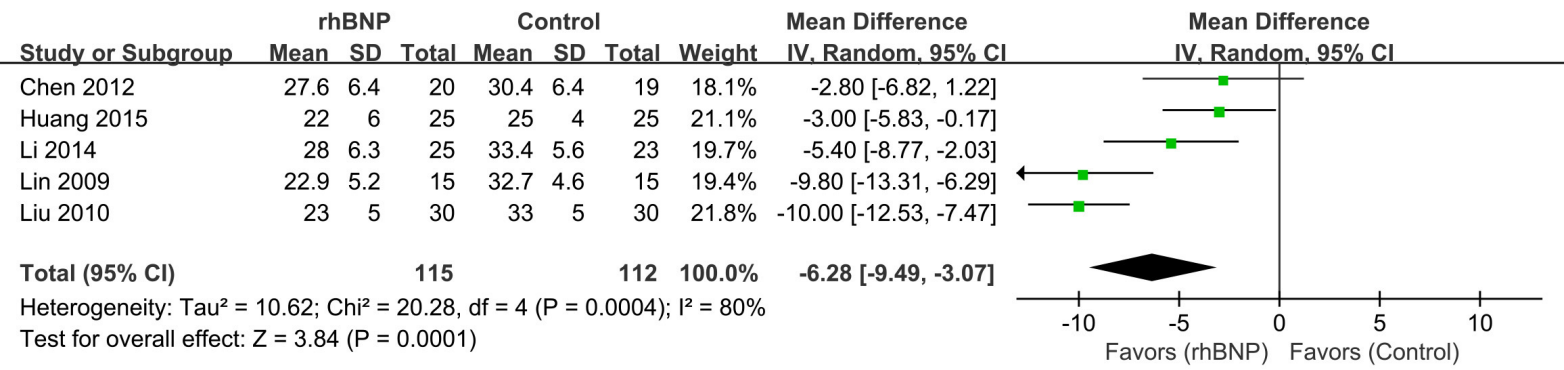
**Comparison of pulmonary artery pressure (PAP)**. rhBNP, recombinant human brain natriuretic peptide; SD, standard deviation; df, degrees of freedom.

Five trials provided data on pulmonary artery pressure (Fig. 7). All data displayed heterogeneity (I^2^ = 80%; *p* < 0.001), necessitating the use of a random-effects model. The meta-analysis found a significant decrease in PAP in the rhBNP group compared to the control group (mean difference = –6.28, 95% CI [–9.49, –3.07]; *p* = 0.0001).

### 4.6 Incidence of Adverse Events

Thirteen trials provided information on AEs occurring during hospitalization, encompassing outcomes such as mortality, acute renal failure, the need for dialysis, atrial fibrillation, hypotension, and episodes of dizziness (**Supplementary Fig. 7**). Despite data demonstrating homogeneity (I^2^ = 20%; *p* = 0.24), a random-effects model was employed. The meta-analysis indicated a markedly reduced risk of AEs in patients treated with rhBNP compared to the control group (risk ratio = 0.67, 95% CI [0.51, 0.87]; *p* = 0.002).

### 4.7 The Length of Respiratory Support

Thirteen trials explored the duration of respiratory support (**Supplementary Fig. 8**). These trials exhibited some heterogeneity (I^2^ = 53%; *p* = 0.01), necessitating the application of a random-effects model. The meta-analysis revealed that patients receiving rhBNP experienced a notably shorter duration of respiratory assistance compared to those in the control group (mean difference = –5.07, 95% CI [–6.22, –3.92]; *p* < 0.00001).

### 4.8 ICU LOS

Sixteen trials investigated the ICU LOS, with high heterogeneity (I^2^ = 95%; *p* < 0.00001), leading to the use of a random-effects model (**Supplementary Fig. 9**). The meta-analysis showed a significant decrease in ICU LOS in the rhBNP group compared to the control group (mean difference = –9.39, 95% CI [–13.34, –5.44]; *p* < 0.00001).

### 4.9 Hospital LOS

Eight trials examined the hospitalization LOS (**Supplementary Fig. 10**). These trials exhibited high heterogeneity (I^2^ = 91%; *p* < 0.00001); thus, a random-effects model was utilized. The meta-analysis showed that the administration of rhBNP resulted in a significantly shorter hospital LOS when compared to the control group (mean difference = –2.06, 95% CI [–3.88, –0.23]; *p* = 0.03).

### 4.10 The Maximum Changes in Peak TNF-α Levels

Two trials presented the maximum changes in peak postoperative TNF-α levels (**Supplementary Fig. 11**). A random-effects model was applied, despite the data displaying homogeneity (I^2^ = 0%; *p* = 0.35). The meta-analysis disclosed a markedly significant reduction in peak postoperative TNF-α levels among patients treated with rhBNP compared to the control group (mean difference = –12.89, 95% CI [–18.47, –7.31]; *p* < 0.00001).

## 5. Discussion

The outcomes of our systematic review suggest that administering rhBNP in the perioperative setting can effectively mitigate adverse effects after cardiac surgery, leading to reduced durations of respiratory support, ICU stays, and overall hospitalization. However, rhBNP administration did not reduce postoperative mortality rates among treated patients. Additionally, the results indicated that rhBNP significantly improved LVEF and 24-hour urine volumes, maximal changes in eGFR, reduced pulmonary artery pressure, and peak postoperative SCr and TNF-α levels. These results indicate the potential of administering rhBNP as a key adjunctive therapy in managing the post-cardiac surgery of patients, suggesting a role in improving patient outcomes.

A study by Kolte *et al*. [50] showed that about one-third of patients with severe aortic stenosis with an LVEF <50% experienced improved LVEFs within 1 month after TAVR. Early improvements in LVEF were associated with lower 5-year all-cause mortality and cardiogenic mortality. Since cardiac surgery can improve LVEF, perioperative application of rhBNP and other drugs may encourage more patients to break through the 10% inflection point threshold for LVEF after surgery and improve the prognosis of more patients. Compared to 2022, China has increased the number of cardiac surgery procedures by 78,512, an increase of 29.8% [51], creating a greater challenge in the use and turnover of ICU wards; therefore, reducing the median ICU LOS by 9.39 hours can significantly reduce the pressure on ICU wards and help more patients.

This systematic review found a markedly reduced risk of AEs in patients treated with rhBNP compared to the control group. This risk of AEs was unspecified because of the original articles and included mortality, acute renal failure, the need for dialysis, atrial fibrillation, hypotension, episodes of dizziness, etc. In previous clinical studies on acute decompensated heart failure, hypotension formed the main side effect of rhBNP treatment [52, 53], meaning rhBNP is particularly suitable for patients with hypertensive heart failure. In clinical practice, hypotensive events usually occur about 15 minutes after the rhBNP load dose is applied, while hypotensive events during the rhBNP maintenance dose generally occur less frequently. Therefore, using the maintenance dose may weaken the effect of rhBNP on peripheral vascular dilation, which mainly inhibits the overexcitation of the renin-angiotensin-aldosterone-system and **s**ympathetic nervous system and maintains the arterial blood pressure at a stable level. Stable arterial blood pressure can ensure blood perfusion in the lung, kidney, brain, and other important organs after surgery, and plays an important role in improving the microcirculation of various tissues and organs. The rhBNP loading doses are rarely used in clinical practice unless the patient has high blood pressure before surgery.

Due to the nature of the surgical procedure, we anticipated that all participants would be representative of a broader population scheduled for cardiac surgery and experience a high risk of cardiac complications. We observed minimal evidence of variation in effects across most outcomes. Investigation into the subgroups categorized by the type of surgery did not strongly suggest that these distinctions were likely to impact the findings.

The rhBNP is a synthetic variant of BNP, crafted through recombinant DNA technology, and mirrors the 32-amino acid endogenous BNP in structure and function. This pharmacological mimicry enables rapid relief of symptoms associated with acute decompensated heart failure (ADHF), particularly dyspnea, through mechanisms that reduce preload, afterload, and pulmonary capillary wedge pressure (PCWP) without affecting heart rate [54]. Furthermore, rhBNP dampens neurohormonal activation and positively influences cardiac remodeling [55].

Our meta-analysis demonstrated that administering rhBNP during the perioperative period significantly reduces pulmonary artery pressure and improves LVEF. The rhBNP was found to cause a dose-related decrease in pulmonary-capillary wedge pressure [34]. This effect corresponded to a reduced systemic vascular resistance and an elevation in cardiac index. As rhBNP does not exert a direct inotropic effect on cardiac muscle, the increased cardiac output is likely due to a diminished left ventricular afterload [56]. This protective effect of rhBNP is expected due to its capacity to diminish pulmonary circulation pressure and resistance, augment cardiac output, and enhance systemic perfusion.

Cardiac surgery is a known inducer of multi-organ dysfunction, with acute kidney injury (AKI) being a prevalent complication that extends hospital stays and raises mortality rates. Our meta-analysis revealed that administering rhBNP during the perioperative phase significantly decreased peak SCr levels and enhanced eGFR and 24-hour urine volumes. The natriuretic peptide family potentially protects renal function in patients with heart failure and those undergoing abdominal or cardiac surgery [57, 58, 59]. The latest meta-analysis suggests that BNP is a promising biomarker for acute heart failure (AHF) prognosis; meanwhile, an inverse relationship was identified between eGFR and AHF [60]. Further, rhBNP exerts protective effects by attenuating sympathetic nerve overactivation, reducing circulating norepinephrine levels, suppressing the renin–angiotensin–aldosterone system, and lowering circulating renin/aldosterone levels [61, 62]. The beneficial effects of perioperative rhBNP are likely due to its capacity to bolster cardiac performance, augment renal blood flow, and elevate the glomerular filtration rate.

Moreover, the systemic inflammatory response syndrome (SIRS), a common complication during cardiac surgery, can be triggered by blood contact with the extracorporeal circulation (ECC) system and surgical trauma, adversely affecting patient outcomes. Our meta-analysis included trials that examined the impact of rhBNP on the SIRS, revealing its efficacy in reducing the SIRS marker TNF-α, a key mediator in SIRS pathogenesis. The anti-inflammatory effects of rhBNP involve downregulating proinflammatory cytokines, including TNF-α, by inhibiting IκB phosphorylation, NF-κB expression, and the MAPK pathways [63, 64, 65].

To our knowledge, this study represents the most extensive systematic review to date regarding the impact of rhBNP application on patient outcomes following cardiac surgery. All included trials were RCTs, albeit with some having limited sample sizes. Despite the existence of heterogeneity among the trials, random-effects models were employed to compute the common risk ratio or mean difference for each endpoint. Even when heterogeneity tests were not significant for certain endpoints, such as adverse events and mortality rates, random-effects models were preferred due to their conservative nature compared to fixed models. However, similar results were obtained with both approaches. It is important to acknowledge that the effects of clinical heterogeneity and study design variations cannot be disregarded.

Our findings align with those documented in the study concerning cardiac surgery involving extracorporeal circulation [12]. These findings are also broadly in line with the conclusions from other systematic reviews encompassing RCTs and observational trials [6, 17]. Comparable protective effects have been observed in investigations involving beta-blockers [21, 22] or alpha-2 adrenergic agonists [23] following cardiac surgery, underscoring the importance of the pre-emptive administration in the perioperative period.

Due to the characteristics of the studies included and data availability, these limitations should be mentioned: (1) This study focuses on short-term outcomes during the perioperative period and lacks long-term follow-up data, such as postoperative 30-day mortality and readmission rates. (2) Most of the included studies are of Chinese people (**Supplementary Table 1** shows that most of the studies are from China), which may limit the universality of the conclusions to other races or medical systems. (3) The risk of unspecified AEs, rather than specified AEs, was reported, which cannot provide more detailed information about the safety profile of the treatment.

Future investigations could focus on larger, more diverse populations and explore the long-term outcomes of rhBNP administration in cardiac surgery patients. Further research into the optimal dosage and timing of rhBNP administration could also refine its clinical application, maximizing benefits while minimizing risks.

## 6. Conclusion

In summary, the perioperative use of rhBNP may reduce postoperative complications, abbreviate ICU and hospital LOSs, and potentially attenuate the risk of heart and kidney injury and inflammatory responses following cardiac surgery. Overall, using rhBNP during the perioperative period could offer significant clinical advantages for patients undergoing cardiac surgery.

This systematic review has not been registered. A brief protocol was prepared before we conducted this review, but it was not published. There was no commercial support.

## Availability of Data and Materials

The datasets used and analyzed during the current study are available from the corresponding author on reasonable request.

## Supplementary Material

Supplementary material associated with this article can be found, in the online version, at https://doi.org/10.31083/RCM36423.
