# Supplementary material for: Effects of Perioperative Recombinant Human Brain Natriuretic Peptide in Patients Undergoing Cardiac Surgery: A Systematic Review and Meta-Analysis
Source: Rev Cardiovasc Med. 2025 Sep 18;26(9):36423. doi: 10.31083/RCM36423 (PMC12516758; doi:10.31083/RCM36423)
Supplement: Supplementary file 1 [file 2153-8174-26-9-36423-s1.zip › References for Supplementary Table 1.docx]

[11] Mentzer RM, Jr, Oz MC, Sladen RN, Graeve AH, Hebeler RF, Jr, Luber JM, Jr, *et al*. Effects of perioperative nesiritide in patients with left ventricular dysfunction undergoing cardiac surgery: the NAPA Trial. Journal of the American College of Cardiology. 2007; 49: 716–726. https://doi.org/10.1016/j.jacc.2006.10.048.

[12] Chen HH, Sundt TM, Cook DJ, Heublein DM, Burnett JC, Jr. Low dose nesiritide and the preservation of renal function in patients with renal dysfunction undergoing cardiopulmonary-bypass surgery: a double-blind placebo-controlled pilot study. Circulation. 2007; 116: I134–I138. https://doi.org/10.1161/CIRCULATIONAHA.106.697250.

[13] Lin J. Acute Cardiopulmonary Hemodynamic Effects of Nesiritide in Patients Undergoing Mitral Valve Replacing Surgery. Central South University: Changsha. 2009. (In Chinese)

[14] Liu F, Lin J. Acute Cardiopulmonary Hemodynamic Effects of Nesiritide in Patients Undergoing Mitral Valve Replacing Surgery. Chinese Journal of Thoracic and Cardiovascular Surgery. 2010; 26: 98–99. (In Chinese)

[15] Jiang B. The effects of recombinant human brain natriureticpeptide on the expression of inflammatory factors TNF-α and IL-10 during cardiopulmonary bypass. Central South University: Changsha. 2010. (In Chinese)

[16] Xie L. The Clinical Study about the Influence of Nesiritide on Myocardial Enzymes and NT-proBNP in Patients with Valve Replacement. Central South University: Changsha. 2010. (In Chinese)

[17] Wu J, Wang Y, Li M, Rong X. The effects of recombinant human brain natriuretic peptide on postoperative renal function in patients undergoing cardiac surgery with cardiopulmonary bypass. Journal of Clinical Anesthesia. 2011; 27: 264–266.

[18] Lin G, Luo W, Li Y, Jiang H. The effects of recombinant human brain natriuretic peptide on the plasma endotoxin and systemic inflammatory response in patients with cardiae valve replacement during cardiopulmonary bypass. Chinese Journal of Thoracic and Cardiovascular Surgery. 2012; 28: 294–297. (In Chinese)

[19] Zhang K, Xu D, Shang X, Liu Y, Li H, Liu F, *et al*. Effects of postoperative nesiritide in patients with left ventricular dysfunction undergoing coronary artery bypass. Beijing Medical Journal. 2012; 34: 555–557. (In Chinese)

[20] Zhao D, Zhu S, Wang C. Recombinant Human B-type Natriuretic Peptides in Cardiac Surgical Procedures. Chinese Journal of Clinical Medicine. 2012; 19: 354–355. (In Chinese)

[21] Chen T. Pharmacodynamic Research of Brain Natriuretic Peptide in Treatment of Pulmonary Hypertension after Mitral Valve Replacement. Tianjin Medical University: Tianjin. 2012. (In Chinese)

[22] Gao F, Zhang J, Jiang J, Ding S, Zhou K, Tang K. Influence of recombinant human brain natriuretic peptide on postoperative hemodynamics in patients with severe valvular heart disease. Medical Journal of National Defending Forces in Southwest China. 2013; 23: 833–835. (In Chinese)

[23] Gong S, Wang F, Zhang Z. Effects of recombinant human brain natrinretic peptide on postoperative heart and renal function in patients with renal insufficiency undergoing cardiac surgery with cardiopulmonary bypass. Chinese Journal of Cardiovascular Research. 2014; 12: 553–555. (In Chinese)

[24] Li Z, Wang J, Zhao S, He Z, Qi X, Huang C, *et al*. Effects of recombinant human brain natriuretic peptide on patients with pulmonary hypertension after mitral valve surgery. ACTA UNIVERSITATIS MEDICINALIS NANJING (Natural Science). 2014; 34: 1527–1530. (In Chinese)

[25] Huang Y, Wei Y, Li Y. Effect of Nesiritide on Pulmonary Artery Hemodynamics after Cardiac Valve Replacement. Pharmaceutical and Clinical Research. 2015; 23: 112–113+126.

[26] Jiang Q, Xiang B, Yu T, Huang K., Effectiveness of Preoperative Recombinant Human Brain Natriuretic Peptide on Rheumatic Valves Diseases with Cardiorenal Syndrome: A Randomized Controlled Trial. Chinese Journal of Clinical Thoracic and Cardiovascular. 2016; 23: 760–764. (In Chinese) https://doi.org/10.7507/1007-4848.20160183.

[27] Rong X, Xu Y, Sheng Lin, Jing Z, Wei L, Li M. Influences of recombinant human brain natriuretic peptide on plasma N-terminal pro-brain natriuretic peptide levels and hemodynamics in patients with postoperative pulmonary hypertension. Journal of Clinical Medicine in Practice. 2016; 20: 5–7+14. (In Chinese)

[28] Zhang B, Zheng Y, Fang L, Dai H, Zhu D, Kong Y. The clinical research of rh-BNP on the hemodynamics in patients with dilated cardiomyopathy and severe mitral regurgitation who underwent mitral valve replacement. Journal of Pathology: Clinical Research. 2016; 36: 1750–1753. https://doi.org/10.3978/j.issn.2095-6959.2016.11.012.

[29] Han Q, Zhang H, Wang X. Clinical study on myocardial protective effect of rh-BNP ischemic postconditioning in heart valve replacement. Chongqing Medical Journal. 2016; 45: 4542–4546. (In Chinese)

[30] Wei L, Jiao G, Li M. The investigation of recombinant human brain natrinretic peptide in patients with left ventricular dysfunction undergoing coronary artery bypass grafting with cardiopulmonary bypass. Jilin Medical Journal. 2017; 38: 431–433. (In Chinese)

[31] Wang Y. The Effect of Recombinant Human Brain Natriuretic Peptide to Cardiac Function of Postoperativ Patients with Off-pump Coronary Artery Bypass Graft. Hebei Medical University: Shijiazhuang. 2017. (In Chinese)

[32] Wu X. Effects of freeze-dried recombinant human brain natriureticpeptide on left ventricular function and left ventricularremodeling after CABG. Hebei Medical University: Shijiazhuang. 2017. (In Chinese)

[33] Beaver TM, Cobb JA, Koratala A, Alquadan KF, Ejaz AA. Nesiritide modulates inflammatory response during cardiac surgery: a pilot study. Research in Cardiovascular Medicine. 2018; 7: 137–143.

[34] Xu Y, Li Y, Bao W, Qiu S. Protective effects of recombinant human brain natriuretic peptide in perioperative period during open heart surgery. Experimental and Therapeutic Medicine. 2018; 15: 2869–2873. https://doi.org/10.3892/etm.2018.5750.

[35] Ding S. Effect of rhBNP on Ventricular Remodeling and Hemodynamics after Heart Valve Replacement in Rheumatic Heart Disease. CHINESE J. DIAL. & ARTIF. ORGANS. 2018; 29: 1–2+7.

[36] Song S, Wang Y, Zhou J, Bai C, Yang M., Effects of recombinant human natriuretic peptide on the postoperative BNP,cTnI and LVEF of off-pump coronary artery bypass patients. Journal of Hebei Medical University. 2018; 39: 150–154. https://doi.org/10.3969/j.issn.1007-3205.2018.02.007.

[37] Rong X, Xu Y, Sheng Lin, Jing C, Wei L, Jiao G, *et al*. Effects of intravenous infusion of recombinant human brain natriuretic peptide on cardiac function and serum creatinine level after coronary artery bypass. Guizhou Medical Journal. 2018; 42: 676–677. (In Chinese)

[38] Wang Q. The Effect of Recombinant Human Brain Natriuretic Peptide on Early Cardiorenal Function in Severe Rheumatic Cardiac Valve Disease Patients after Surgery. Modern Diagnosis and Treatment. 2020; 31: 1723–1725.

[39] Zhao J. Effects of Recombinant Human Brain Natriuretic Peptide Pretreatment on Cardiac Function in Patients undergoing Valve Replacement. University of Electronic Science and Technology of China: Chengdu. 2021. (In Chinese)

[40] Liu H, Guo S, Wang Y, Song Y. Perioperative analysis of recombinant human brain natriuretic peptide in patients undergoing cardiac valve replacement with low left ventricular ejection fraction value. Chinese Heart Journal. 2022; 34: 325–328. https://doi.org/10.12125/j.chj.202111016.

[41] Bu L. Effect of Xinhuosu on early prognosis of patients undergoing cardiac valve replacement under cardiopulmonary bypass. China Practical Medical. 2023; 18: 13–16. (In Chinese) https://doi.org/10.14163/j.cnki.11-5547/r.2023.08.003.

[42] Chen L. Application of freeze-dried recombinant human brain natriuretic peptide in perioperative period of cardiac surgery. Chengde Medical University: Chengde. 2023. (In Chinese)

[43] Pan Y. Application of Recombinant Human Brain Natriuretic Peptide in Patients after Off-pump Coronary Artery Bypass Grafting. Chinese and Foreign Medical Research. 2023; 21: 125–128. (In Chinese)

[44] Ejaz AA, Martin TD, Johnson RJ, Winterstein AG, Klodell CT, Hess PJ, Jr, *et al*. Prophylactic nesiritide does not prevent dialysis or all-cause mortality in patients undergoing high-risk cardiac surgery. The Journal of Thoracic and Cardiovascular Surgery. 2009; 138: 959–964. https://doi.org/10.1016/j.jtcvs.2009.05.014.

[45] Beaver TM, Winterstein A, Hess PJ, Jr, Martin TD, Arnaoutakis GJ, Peng YG, *et al*. Nesiritide following maze and mitral valve surgery. Journal of Cardiac Surgery. 2008; 23: 431–436. https://doi.org/10.1111/j.1540-8191.2007.00552.x.

[46] Florida UO. Does Nesiritide Provide Renal Protection. ClinicalTrial.gov. 2010. Available at: https://clinicaltrials.gov/ct2/show/NCT01440881 (Accessed: 21 May 2024).

[47] Costello JM, Dunbar-Masterson C, Allan CK, Gauvreau K, Newburger JW, McGowan FX, Jr, *et al*. Impact of empiric nesiritide or milrinone infusion on early postoperative recovery after Fontan surgery: a randomized, double-blind, placebo-controlled trial. Circulation. Heart Failure. 2014; 7: 596–604. https://doi.org/10.1161/CIRCHEARTFAILURE.113.001312.

[48] Costello JM. Nesiritide Use Following Cardiac Surgery in Infants. ClinicalTrial.gov. 2006. Available at: https://clinicaltrials.gov/ct2/show/NCT00281671 (Accessed: 21 May 2024).
