# Supplementary material for: Effects of Perioperative Recombinant Human Brain Natriuretic Peptide in Patients Undergoing Cardiac Surgery: A Systematic Review and Meta-Analysis
Source: Rev Cardiovasc Med. 2025 Sep 18;26(9):36423. doi: 10.31083/RCM36423 (PMC12516758; doi:10.31083/RCM36423)
Supplement: Supplementary file 1 [file 2153-8174-26-9-36423-s1.zip › Supplementary material.docx]

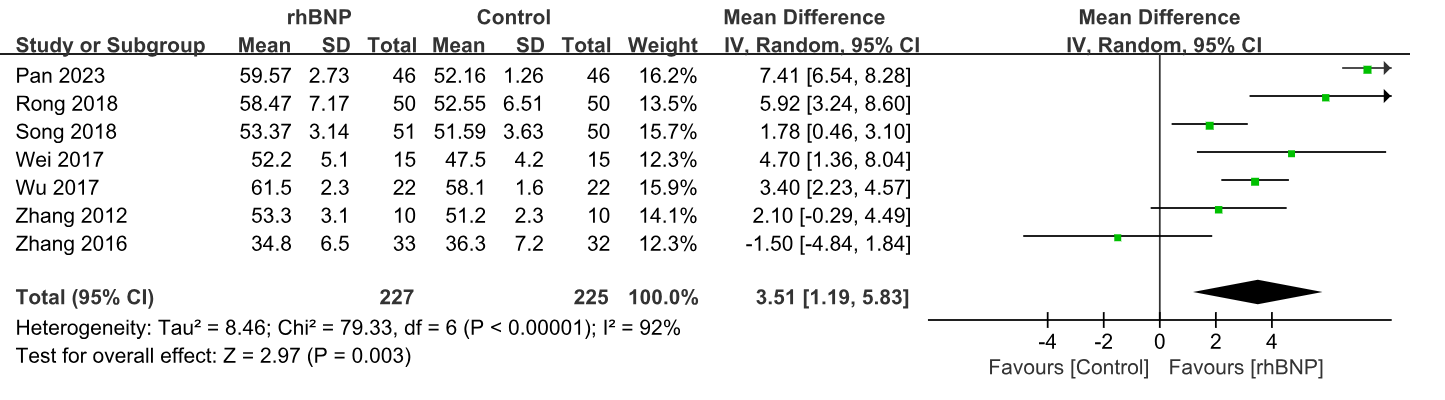


Supplementary Fig. 1. Comparison of left ventricular ejection fraction (LVEF) within patients using CABG. rhBNP, recombinant human brain natriuretic peptide; SD, standard deviation; df, degrees of freedom.


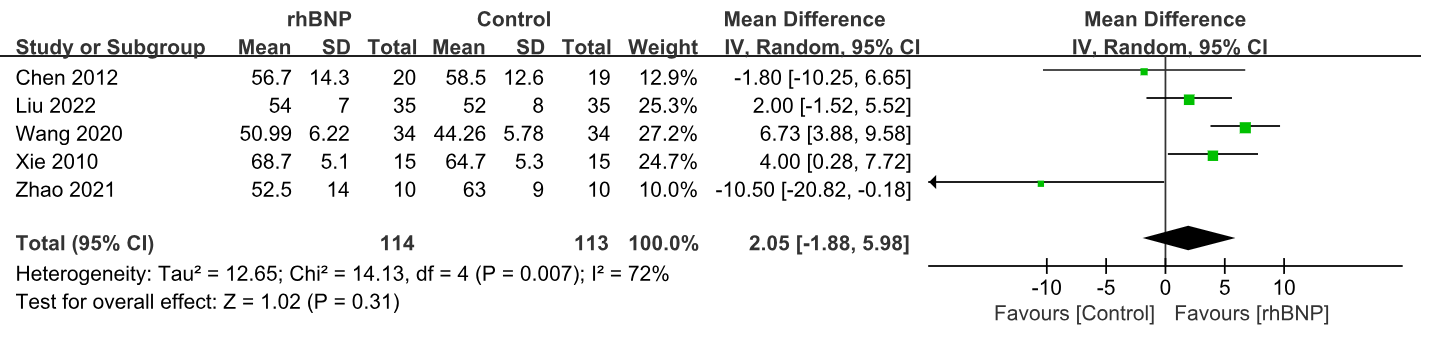


Supplementary Fig. 2. Comparison of left ventricular ejection fraction (LVEF) within patients using valve procedure. rhBNP, recombinant human brain natriuretic peptide; SD, standard deviation; df, degrees of freedom.


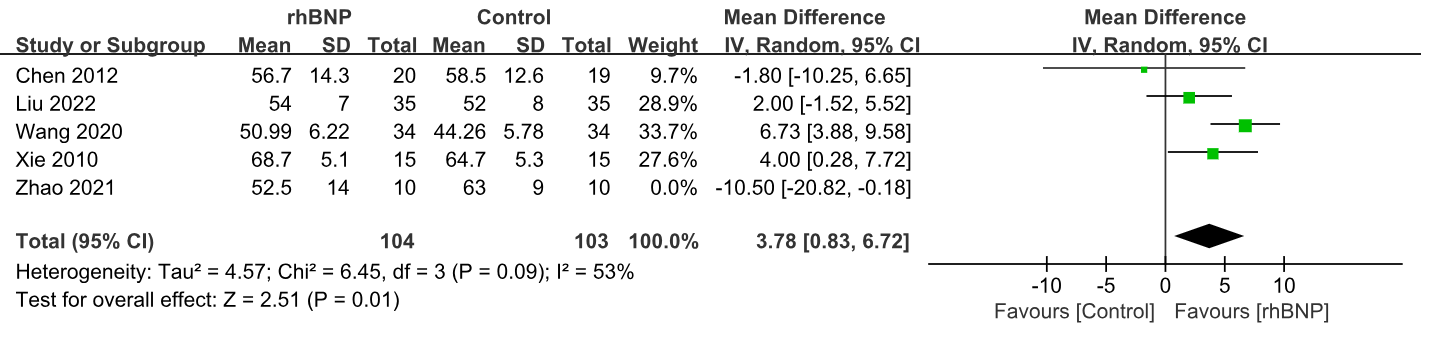


Supplementary Fig. 3. Comparison of left ventricular ejection fraction (LVEF) within patients using valve procedure excluding Zhao 2021. rhBNP, recombinant human brain natriuretic peptide; SD, standard deviation; df, degrees of freedom.


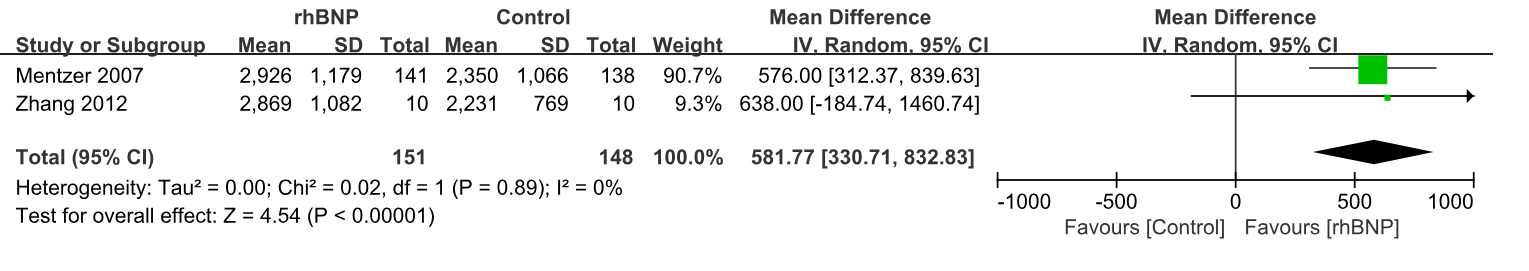


Supplementary Fig. 4. Comparison of 24-hour urine volumes within patients using CABG. rhBNP, recombinant human brain natriuretic peptide; SD, standard deviation; df, degrees of freedom.


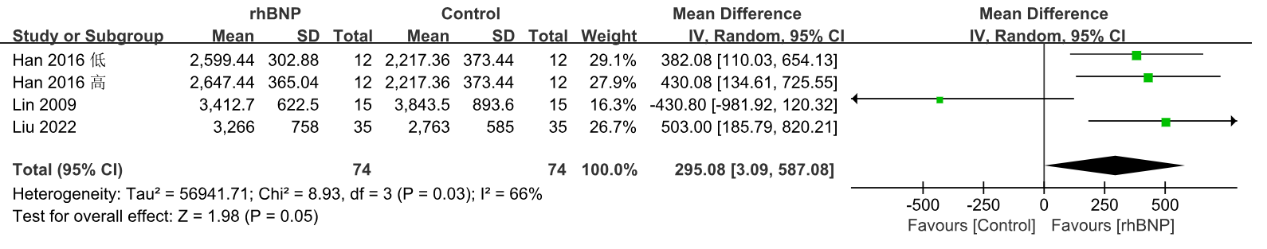


Supplementary Fig. 5. Comparison of 24-hour urine volumes within patients using valve procedure. rhBNP, recombinant human brain natriuretic peptide; SD, standard deviation; df, degrees of freedom.


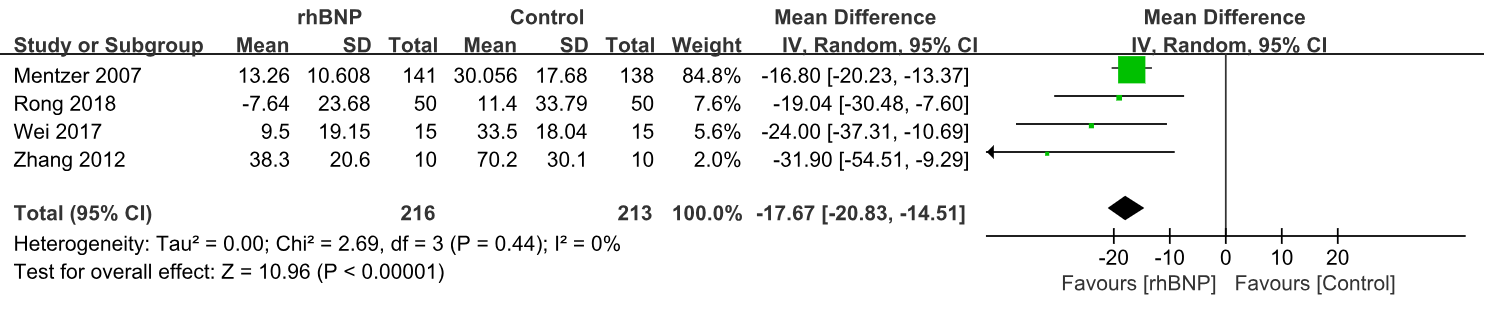


Supplementary Fig. 6. Comparison of serum creatinine (Scr) within patients using CABG. rhBNP, recombinant human brain natriuretic peptide; SD, standard deviation; df, degrees of freedom.


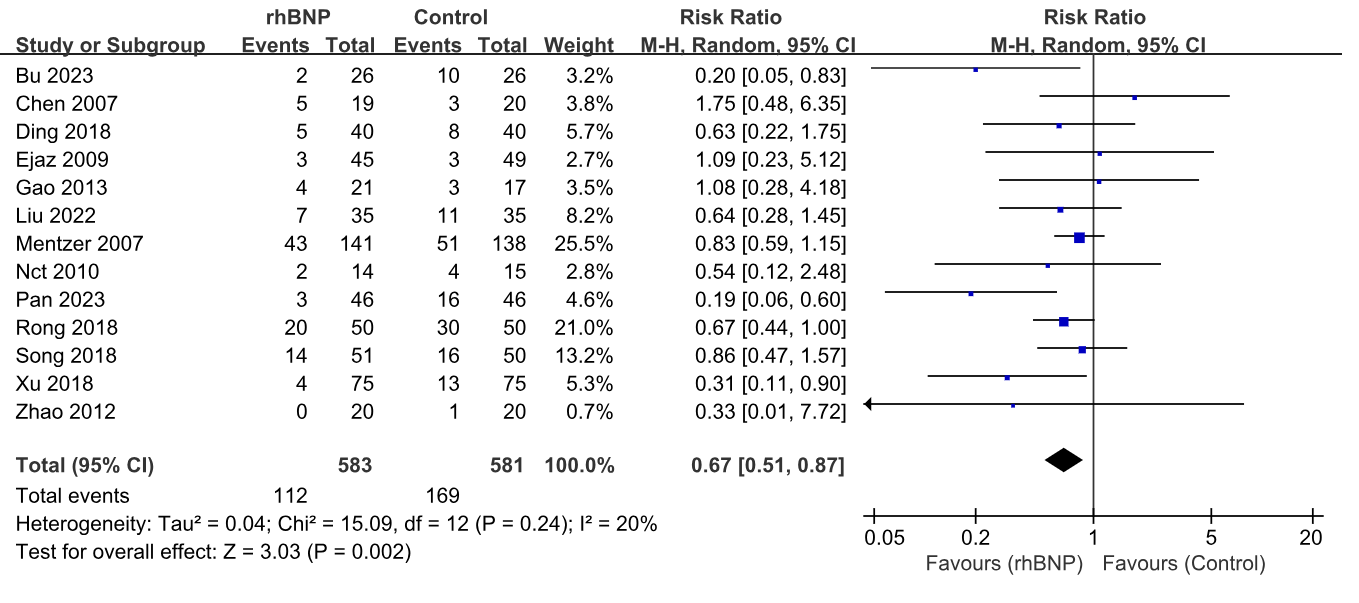


Supplementary Fig. 7. Comparison of incidence of adverse event (AE). rhBNP, recombinant human brain natriuretic peptide; df, degrees of freedom.


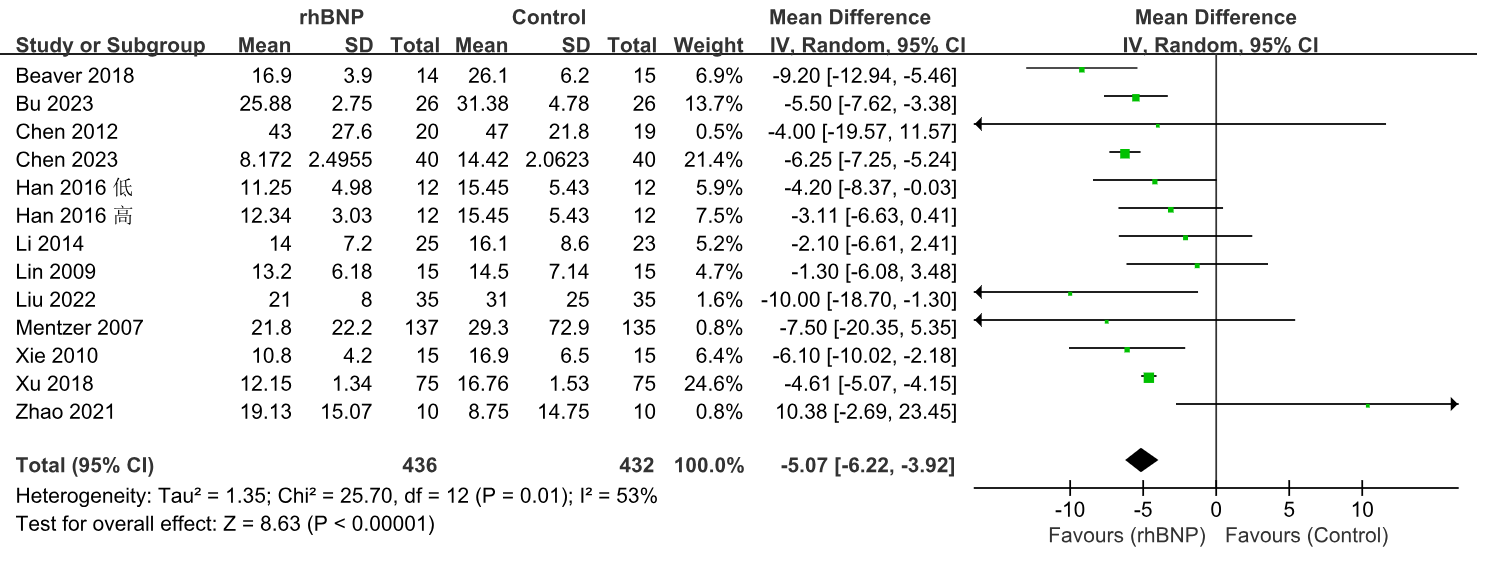


Supplementary Fig. 8. Comparison of the length of respiratory support. rhBNP, recombinant human brain natriuretic peptide; SD, standard deviation; df, degrees of freedom.


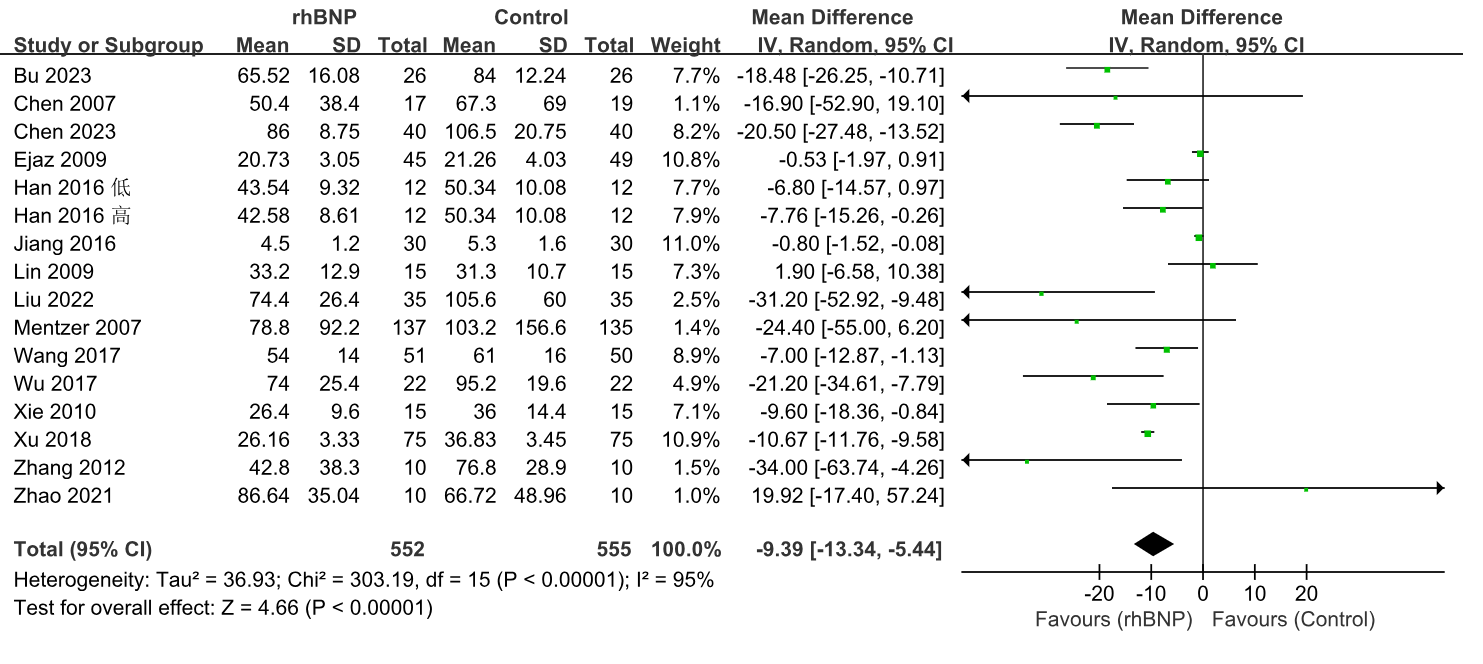


Supplementary Fig. 9. Comparison of duration of ICU stay. rhBNP, recombinant human brain natriuretic peptide; SD, standard deviation; df, degrees of freedom.


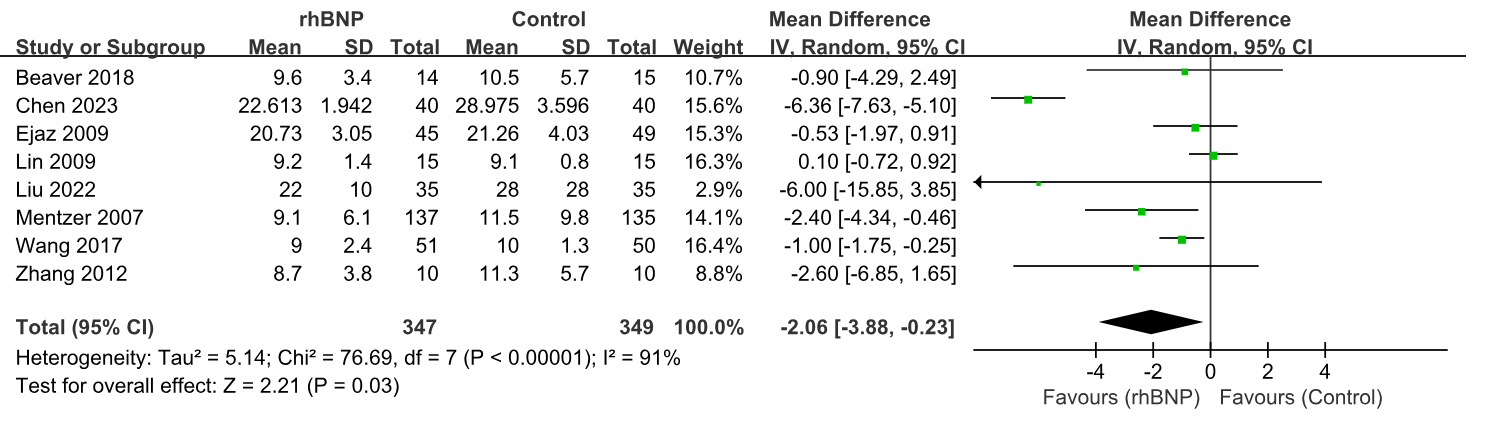


Supplementary Fig. 10. Comparison of duration of hospitalization. rhBNP, recombinant human brain natriuretic peptide; SD, standard deviation; df, degrees of freedom.


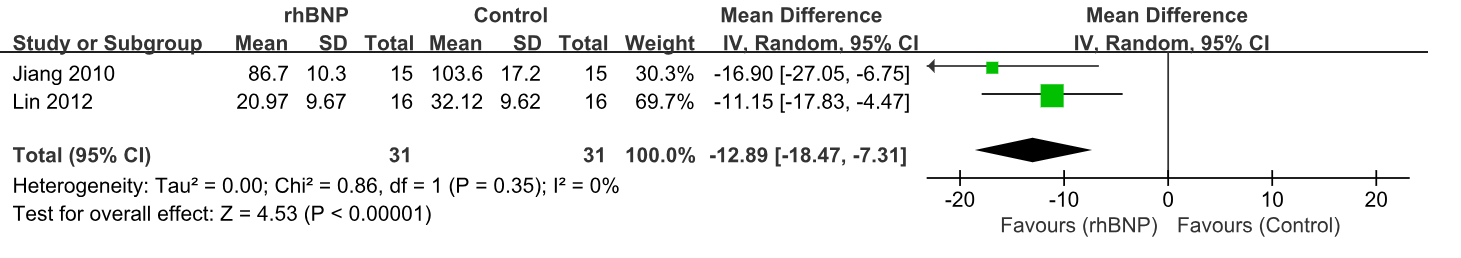


Supplementary Fig. 11. Comparison of the maximum changes in peak postoperative levels of TNF-α. rhBNP, recombinant human brain natriuretic peptide; SD, standard deviation; df, degrees of freedom.


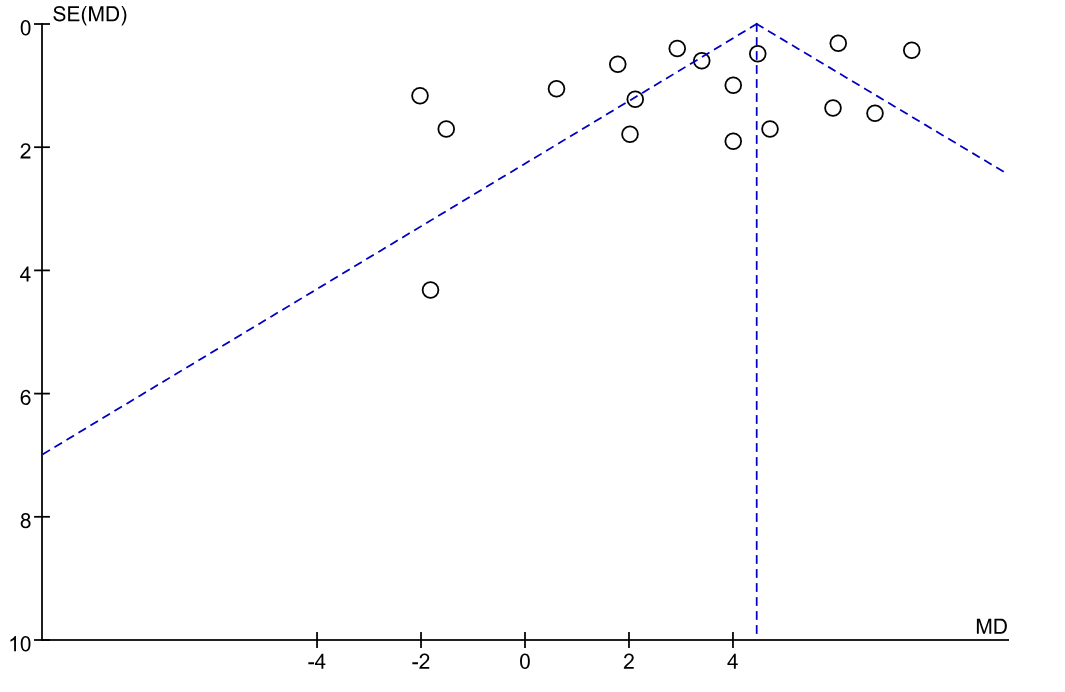


Supplementary Fig. 12. Funnel plot of comparison of left ventricular ejection fraction (LVEF). MD, mean difference. SE, standard error.


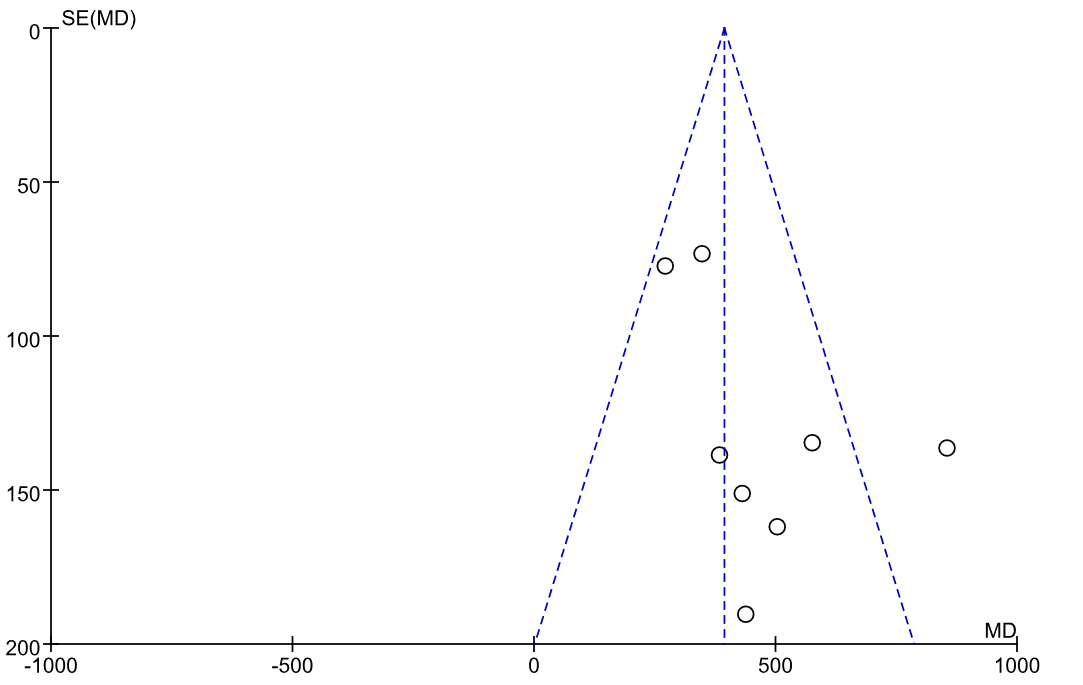


Supplementary Fig. 13. Funnel plot of comparison of 24-hour urine volumes. MD, mean difference. SE, standard error.


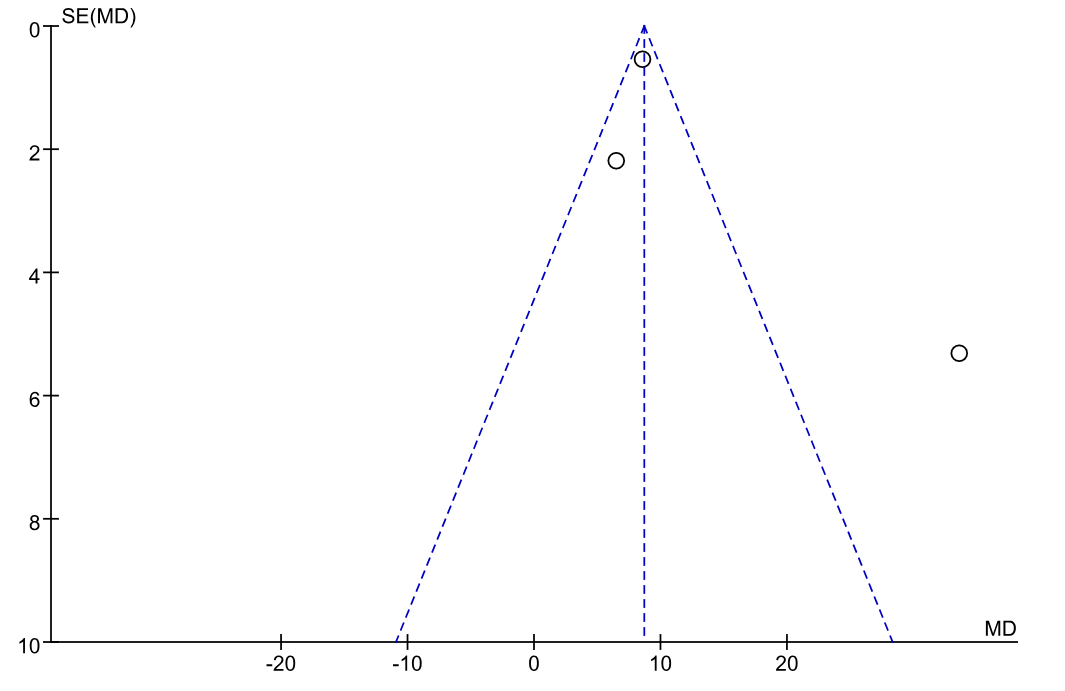


Supplementary Fig. 14. Funnel plot of comparison of estimated glomerular filtration rate (eGFR). MD, mean difference. SE, standard error.


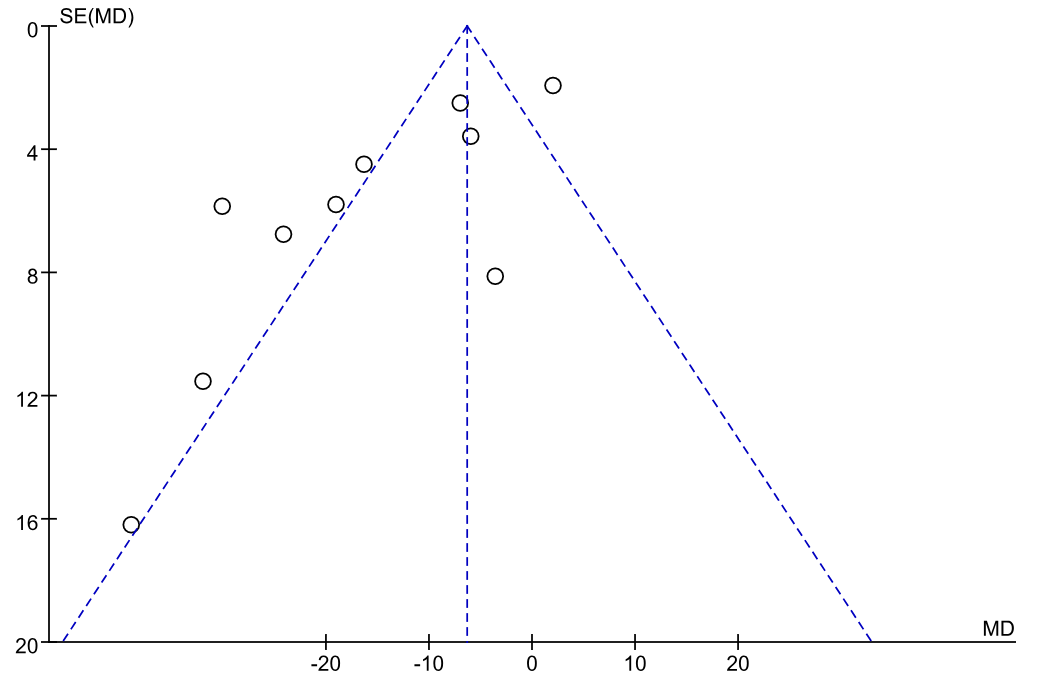


Supplementary Fig. 15. Funnel plot of comparison of serum creatinine (Scr). MD, mean difference. SE, standard error.


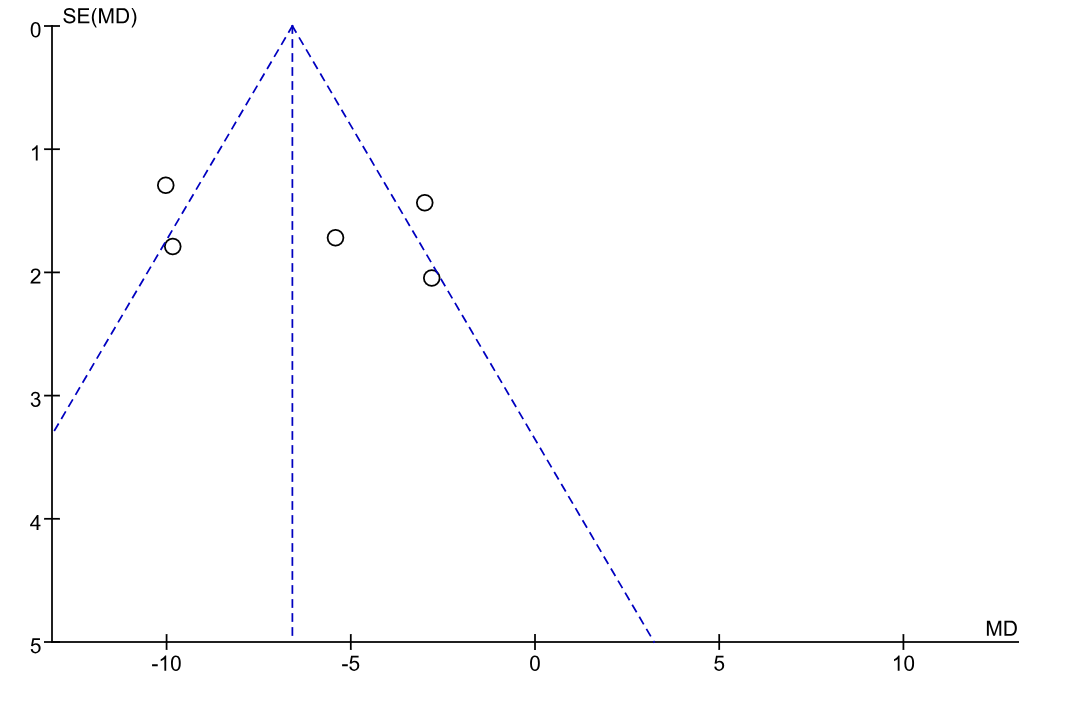


Supplementary Fig. 16. Funnel plot of comparison of pulmonary artery pressure (PAP). MD, mean difference. SE, standard error.


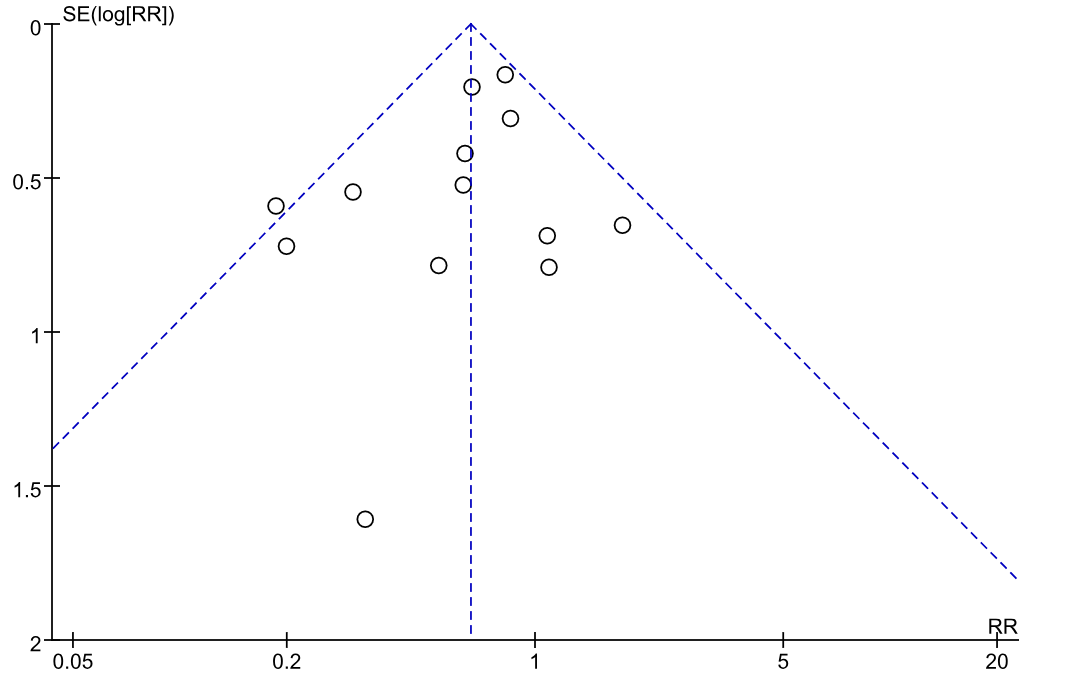


Supplementary Fig. 17. Funnel plot of comparison of incidence of adverse event (AE). RR, risk ratio. SE, standard error.


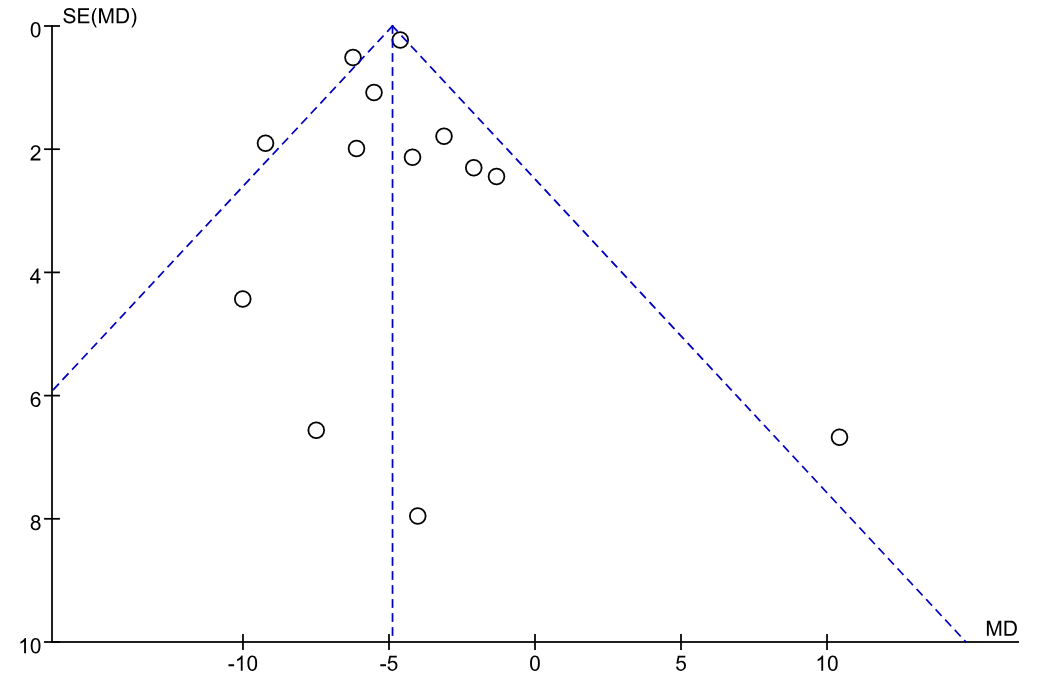


Supplementary Fig. 18. Funnel plot of comparison of the length of respiratory support. MD, mean difference. SE, standard error.


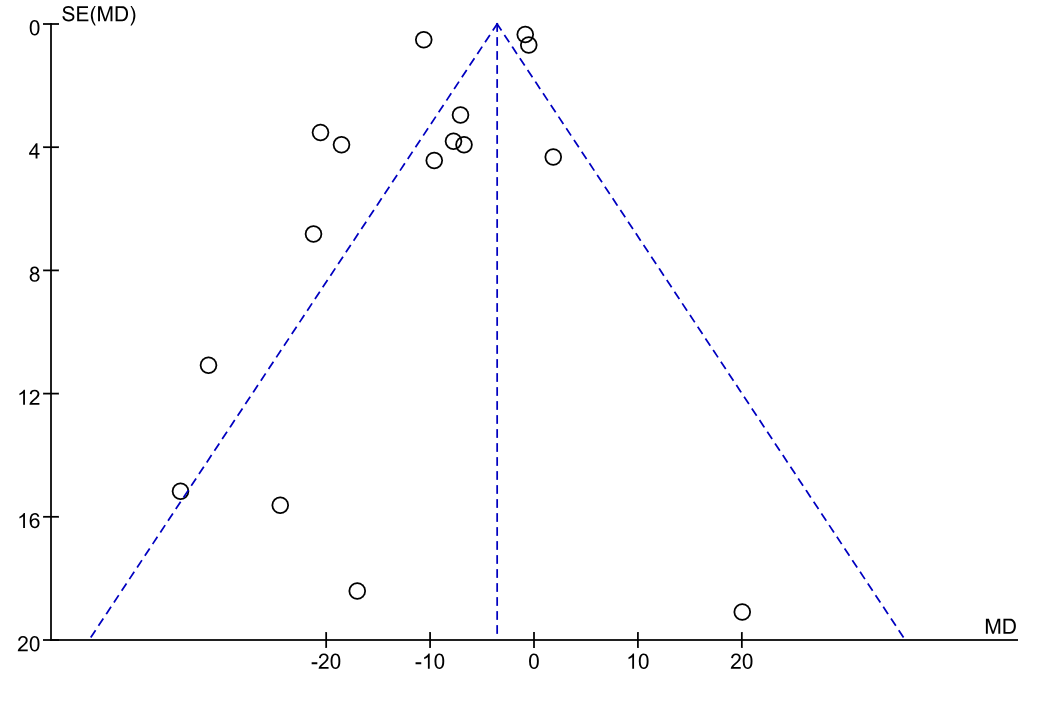


Supplementary Fig. 19. Funnel plot of comparison of duration of ICU stay. MD, mean difference. SE, standard error.


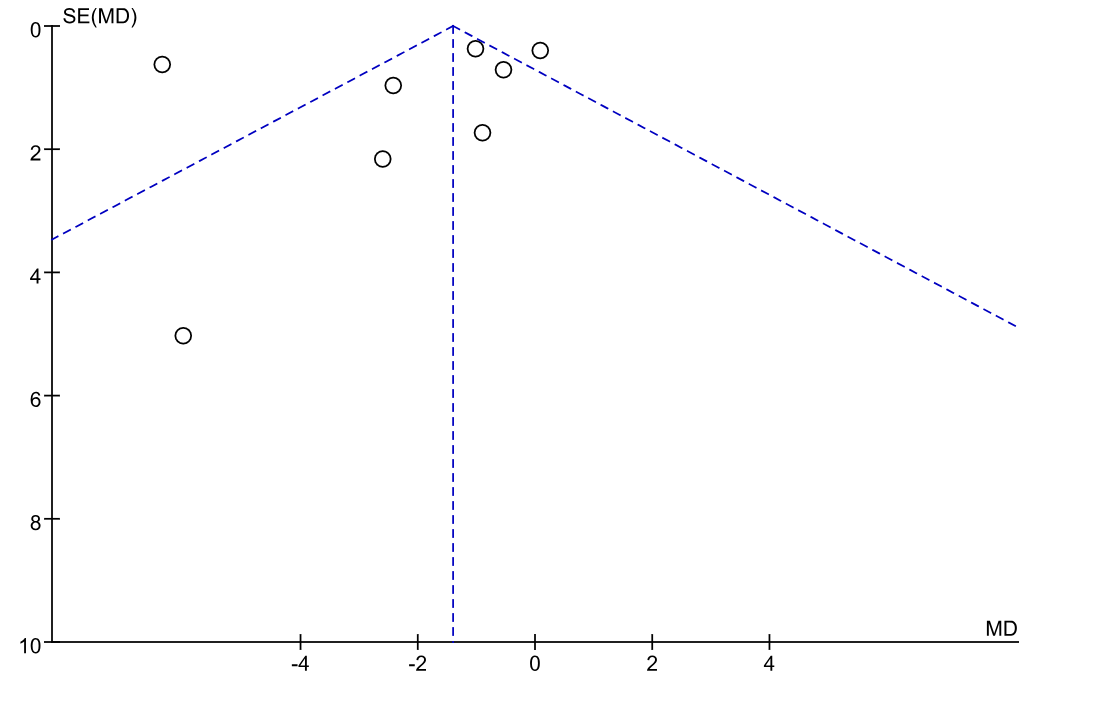


Supplementary Fig. 20. Funnel plot of comparison of duration of hospitalization. MD, mean difference. SE, standard error.


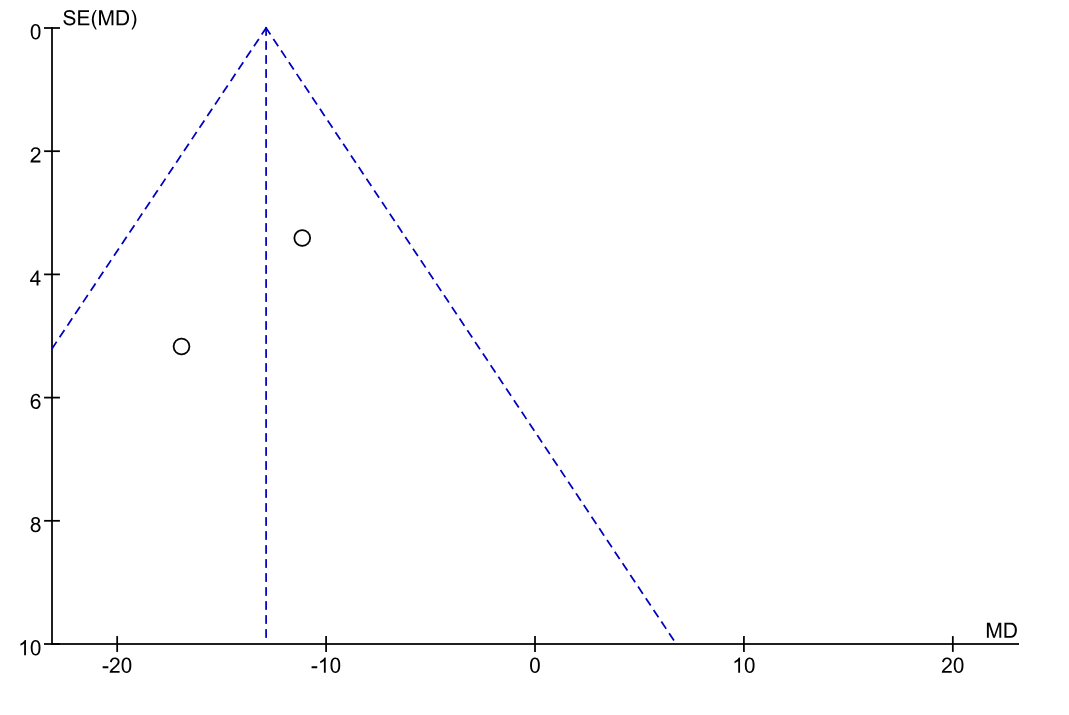


Supplementary Fig. 21. Funnel plot of comparison of maximum changes in peak postoperative levels of TNF-α. MD, mean difference. SE, standard error.
